# Crafts as an example of active and productive aging: profiles of artisans and family transmission in a UNESCO Creative City of Crafts and Folk Art

**DOI:** 10.3389/fpsyg.2025.1651086

**Published:** 2025-08-29

**Authors:** Sandra Igreja, Soraia Teles, Constança Paúl

**Affiliations:** RISE-Health, Department of Behavioral Sciences, School of Medicine and Biomedical Sciences, University of Porto (ICBAS-UP), Porto, Portugal

**Keywords:** older adults, crafts, folk art, active aging, work, family transmission

## Abstract

In light of global demographic changes, sustainable solutions promoting active and healthy aging are crucial. Crafts, besides preserving cultural heritage, offer a meaningful occupation. Barcelos (Portugal), a UNESCO Creative City of Crafts and Folk Art, stands out for its strong craft activity, predominantly by older artisans. However, studies addressing their profiles, family transmission dynamics, and craftsmanship as an example of active aging are lacking, which this study aims to explore. An observational cross-sectional study was conducted, with primary data collection through on-site questionnaires at the ateliers. A non-probability sample of older artisans (aged 55 or older) from different craft sectors was recruited. The participants (*n* = 55) were mostly male (60%, *n* = 33), with a mean age of 67.5 years (SD = 8.02) and 6.4 years of formal education (SD = 3.32). They worked across eight craft and folk art sectors, with the Imagery sector being the most represented (52.7%). A weekly average of 51 h (SD = 17.05) dedicated to the activity was reported. Most participants started in crafts before the age of 20 (76.3%; *n* = 42), and 60% (*n* = 33) continued the craft activity after retirement. The activity was transmitted by family members from previous generations (63.6%; *n* = 35), and 70.9% (*n* = 39) had family members involved in the activity; among those practicing the craft with other or same-generation relatives (*n* = 34), 58.8% engaged in the craft with collateral or affinal relatives. Most artisans reported that the activity began in the family three or more generations ago and dates to the 19th century. This study makes an original contribution to gerontological research, highlighting craftsmanship as an example of active and productive aging. It informs public policy discussions supporting older artisans, fostering intergenerational craft transmission, family business sustainability, and cultural heritage preservation.

## 1 Introduction

Over decades, a vast body of evidence has accumulated on the factors that contribute to active aging in the workplace, including individual, professional, team-related, organizational, extra-work, and social dimensions (Zacher et al., 2018). The active aging approach has the potential to enable countries to successfully respond to the challenges of an aging population due to its comprehensive focus and emphasis on both social and individual responsibility (Foster and Walker, 2015). It must be recognized that there are several pathways for older adults to age actively (Boudiny, 2013). Aging implies that the role of older individuals is not limited to physical and occupational activities alone but involves, among other things, participation in social, economic, and cultural processes. Productive activities hold significant meaning for the individuals and create social value, whether paid or unpaid (Vega-Tinoco et al., 2022). Various factors shape the meaning of work, including the social and environmental contexts in which it develops (Rosso et al., 2010). Previous studies have observed that there may be benefits for older adults in prolonging professional activity, with positive effects on health, life satisfaction, and quality of life (Chang and Yen, 2011; Costa et al., 2018; Baxter et al., 2021). The fundamental rhetoric of active aging is the recognition of the autonomy and capacity of older citizens to engage in meaningful social actions, as opposed to disengagement, and the removal of age-related barriers to participation in the labor market. This concept paves the way for a new legitimacy for what is considered successful aging, which largely depends on almost endless participation in the productive sphere of society (Paúl and Lopes, 2016).

Older adults often value their involvement in traditional forms of artistic production, an occupation that promotes well-being in various dimensions and contributes to the preservation and transmission of skills in art and crafts (Tzanidaki and Reynolds, 2011; Lee and Heo, 2021). Previous studies have found that roles perceived as highly valuable were those of family members (Park and Lee, 2022). The roles played by older generations are relevant from the perspective of adult development, being part of processes of generativity and legacy creation linked to health and well-being (Ashida and Schafer, 2015; Shahen et al., 2019; Bower et al., 2021).

According to Erikson’s psychodynamic perspective, human development and, in particular, the achievement of identity continues into the final years of life; the resolution of successive crises reinforce the construction of identity (Jesuíno, 2012). Older adults and their families face challenges that are as complex and rich as those encountered by younger generations (King and Wynne, 2004). Alongside the roles occupied in the work domain, the family sphere, and the roles therein, constitutes the most important factor in defining each individual’s happiness across the life course (Lopes and Gonçalves, 2012). The concept of family integrity refers to the positive, end-point outcome of the older adult’s striving for meaning, connection, and continuity within the multigenerational family context (King and Wynne, 2004). Culture and creative activity can facilitate aging and help develop intergenerational relationships. In old age, individuals may discover the desire to explore new creative, emotional, and relational spaces and creativity enhances the lives of many older adults (Cristini and Cesa-Bianchi, 2019).

Creativity has been recognized as a relevant mechanism for self-realization in older adults (Gallistl, 2018), and engagement in creative activities, such as crafts, has been associated with benefits to subjective well-being (Keyes et al., 2024), reinforcing the importance of such practices as means of personal fulfillment and the promotion of active aging. This study focuses on the experiences of older artisans in the city of Barcelos (Portugal), which has been designated by UNESCO as a Creative City of Crafts and Folk Art (Município de Barcelos, 2023; UNESCO, 2024).

Despite the reported benefits and the sociocultural and economic relevance of this activity, comprehensive studies describing the sociodemographic and professional profiles of artisans practicing in this Creative City are lacking, as is research examining the family transmission of the craft. Therefore, this study aims to describe the sociodemographic and professional profiles of a sample of older artisans from Barcelos and to analyze family transmission dynamics in crafts, while framing this practice as an example of active and productive aging.

## 2 Crafts and folk art: the legacy of Barcelos, Portugal

The world of crafts has always been conceptually difficult to define (Pereira, 2024). Traditional crafts move across various discursive frameworks of creation and practice (including economic development, design, the valuing of memory and ancestral knowledge, and the transition from folk art to contemporary art). Crafts are often described as the result of manual labor combined with the artisan’s technical precision and creative freedom (Reis, 2022). The production of material objects and artifacts seemingly follows two directions: on one hand, an increasing production of utilitarian items; on the other, a deepening of craft practices with symbolic, ritualistic, and magical dimensions, marked by significant autonomy (Pereira, 2024). In general, the history of crafts is the history of civilization itself, beginning with the earliest use of tools to create material objects. It is a form of work that requires a high degree of manual dexterity and/or artistic skill. Crafts are increasingly associated with economic recovery (Jakob, 2013).

The theme of folk art in Portugal emerged alongside the rise of European ethnology in the late 19th century. Along with literature and popular traditions, it became one of the main fields in which the ethnological tradition developed, often linked to projects of thematizing national identities. The first approaches to folk art came from the field of art history, with a central figure being Vasconcelos, whose reflections emphasized two main aspects, both shaped by the relationship between what is considered popular and what is considered national: on the one hand, the defense of a close connection between certain expressions of folk art (such as the Minho yokes and Romanesque as a national style), and on the other, his role as a promoter of *home industries* (Vasconcelos, 1918; Leal, 2024). While it began within art history, this interest in folk art quickly extended into ethnography, particularly through the work of Peixoto (1990), as discussed in Leal (2024).

There is no clear boundary separating crafts from art; rather, they form a craft-art continuum that is not built along a single line but interwoven through multiple lines of thought and practice (Buszek, 2011). Beyond being a professional activity, crafts play a key role in sustaining tradition and preserving cultural heritage (Lumpkin and Brigham, 2011; Deb et al., 2022). Many people invest emotionally in these activities and continue practicing them throughout all stages of life (Kenning, 2015).

Art and craft as family enterprises are distinct from other types of family businesses and entrepreneurial activities as the nature of these businesses involves more factors. In the family craft business, economic, promotional, motivational, support and challenge factors are considered indicators of success for their adoption (Deb et al., 2022).

In Barcelos (Portugal), the commitment to crafts is evident: “It is the older artisans who embody this realism, and they are the only ones who do it all. From them, the transmission of routine knowledge is expected, along with stories and explanations, the transmission of myths, and their continuous recreation with the freedom they derive from life’s experiences” (Costa, 1991, n.p.; Costa, 2024, p. 145). The territory is distinguished by its extensive craft activity, with a natural predominance of Pottery and Imagery. Throughout the 19th century, there are numerous references to the pottery produced in the region, and its ateliers became nationally recognized (Correia, 1965). As for Imagery, it “encompasses all pieces that are, in general, decorative in purpose and rooted in popular modeling. Here we find a world of forms: the full range of animals known to the clay sculptors, regional characters and customs, popular, political, and religious scenes, images, satires, caricatures, and oddities, etc. It is also of great antiquity, and it is impossible to determine its origins” (Correia, 1965, pp. 22, 24). A study from 1899 already presented detailed information about these crafts made in the region, including a description of the themes represented, among which there was the Barcelos rooster (Correia, 1965; Peixoto, 1990). The same study references the fairs, showing the historical and economic relevance of these spaces in promoting these products of Barcelos (Peixoto, 1990).

The Imagery of Barcelos is mentioned by the Nobel laureate of Portuguese literature as the land of the rooster and the legion of clay (Saramago, 1990). The Barcelos Rooster (Galo de Barcelos) and the extensive creation of figurines have inspired a dialogue between the realms of folk culture and the worlds of art (Almeida, 2024). Barcelos has built an important sociocultural and economic heritage around the tradition of crafts and folk art. The municipality has valued and protected this heritage, being the only region in Portugal with three certified artisanal productions: Pottery of Barcelos, Imagery of Barcelos, and the Crivo Embroidery of São Miguel da Carreira. The region’s ateliers cover various sectors of crafts and folk art, including, in addition to Imagery, Pottery and Embroidery, Weaving, Iron and Derivatives, Wood, Basketry and Wicker, and Contemporary Crafts. This context led Barcelos to be recognized by UNESCO as a Creative City of Crafts and Folk Art, becoming the first municipality in Portugal to join the UNESCO Creative Cities Network in the field of crafts and folk art (Comissão Nacional da UNESCO, 2023; Município de Barcelos, 2023; UNESCO, 2024).

## 3 Materials and methods

### 3.1 Study design

An observational, cross-sectional study was conducted, with primary data collected in person through a survey specifically designed for this study and administered at the ateliers of older professional artisans. This approach was considered appropriate for the study’s objectives, as it allows for the description of the sociodemographic and professional profiles of the participants, as well as the analysis of family transmission dynamics in craftwork and the exploration of associations between these variables.

### 3.2 Participants and recruitment

A non-probabilistic sample of professional artisans working in the municipality of Barcelos was recruited for this study. Eligibility criteria included individuals aged 55 or older, living in the community (not in institutional care), who were professionally engaged in crafts and folk art, working in ateliers located within the region. Artisans from any of the following sectors were eligible: Imagery, Pottery, Embroidery, Weaving, Iron and Derivatives, Wood, Basketry and Wicker, and Contemporary Crafts. Potential participants were identified through public platforms such as artisans’ personal websites and the official website of the Municipality of Barcelos, which offers information on local craft routes (a list of artisans, with their atelier addresses and contact details) (Município de Barcelos, 2023). A convenience sample was used, aiming to include participants representing the region’s various craft sectors.

After identification, the artisans were contacted, fully informed about the study, and invited to participate. Those who showed interest were assessed for eligibility, and subsequent data collection phases were scheduled with eligible participants. Data collection took place in person at the artisans’ ateliers during the first quarter of 2024. Fifty-five older professional artisans participated in the study, representing 27.4% of all artisans listed in the municipality’s craft routes (*n* = 201).

### 3.3 Instruments

A survey specifically designed to collect sociodemographic information, data on professional activity, and information related to the family transmission of crafts and folk art was administered to the study participants. The questionnaire, interviewer-administered, was conducted in the natural context, directly at the participants’ ateliers.

Older professional artisans were asked to respond to questions related to sociodemographic characteristics, such as age, gender, marital status, years of education and professional training, retirement status, main source of income, housing and economic situation, and number of years living in the locality. The information collected about the professional activity included the craft sector, starting age at the craft activity, whether the atelier is located at the residence, main raw materials, key themes represented, weekly hours dedicated to the craft activity, and the days of the week when the activity is performed. Non-professional activities and the time dedicated to these were also collected. The artisans were asked questions regarding the family transmission of crafts and folk art, including whether the activity was passed down by relatives from previous generations; which family members were involved in transmitting the activity; when the craft activity began in the family; the number of generations involved in the craft and folk art sector up to the present; transmission of the activity to younger generations; whether they have family members working in the sector, and if so, whether they are older and/or younger; regular practice of the activity with family members of different generations.

### 3.4 Data analysis

#### 3.4.1 Descriptive statistical analysis

Descriptive statistics were calculated, including absolute and relative frequencies as well as measures of central tendency and dispersion, selected according to the nature and distribution of the variables.

Parametric and non-parametric tests were chosen, as appropriate based on the data distribution, to explore associations between sociodemographic characteristics, variables related to professional activity, and variables related to family dynamics. The t-test was employed for normally distributed continuous variables and the Mann–Whitney U test for asymmetric continuous variables or ordinal ones. Associations between categorical variables were analyzed using the chi-square test and Fisher’s exact test, as appropriate. Correlations were examined using Pearson’s correlation coefficient or Spearman’s rank correlation coefficient, depending on the nature of the data. The F-statistic was used in ANOVA to compare means across multiple groups. Statistical tests were two-tailed, and the significance level was set at 0.05. Data analysis was performed using SPSS software, version 29.

The reporting of this study follows the Strengthening the Reporting of Observational Studies in Epidemiology (STROBE) statement (von Elm et al., 2007). The STROBE checklist is included in Supplementary material 1.

#### 3.4.2 Ethical approval and data protection

This study is part of a larger project titled “Folk Art in the Quality of Life and Well-being of Older Artisans and Families,” which aims to explore the contribution of artistic activity and the legacy of crafts and folk art to the quality of life, health status, and family cohesion of professional artisans in the municipality of Barcelos, Portugal.

The project was approved by the joint Ethics Committee of the Centro Hospitalar Universitário de Santo António, E. P. E (CHUdSA) and the Instituto de Ciências Biomédicas Abel Salazar, Universidade do Porto (ICBAS-UP) [Ethics Committee CHUdSA/ICBAS], reference 2024/CE/P02(P418/2023/CETI) and by the Data Protection Unit of the University of Porto, reference R-6/2025.

All participants were provided with information about the project and consented to their participation in the research.

## 4 Results

### 4.1 Sociodemographic profile of older artisans

Table 1 describes the sociodemographic profile of the artisans. Participants had a mean age of 67.49 years (SD = 8.02), with an overall age range of 55–88 years. More than half were male (60%, *n* = 33). Among male participants, ages ranged from 55 to 84 years (mean = 67.67; SD = 7.87), and among female participants (*n* = 22), ages ranged from 55 to 88 years (mean = 67.23; SD = 8.42).

**Table 1:** Sociodemographic profile of the artisans.

Variables	*N*	Descriptive statistics
Gender, *n* (%)	55	
Male		33 (60)
Age, M (SD)	55	67.49 (8.02)
Education (years) M (SD)	55	6.42 (3.32)
Marital status, *n* (%)	55	
Married		47 (85.5)
Widow(er)		5 (9.1)
Divorced		2 (3.6)
Single		1 (1.8)
Professional training, *n* (%)	55	
Yes		13 (23.6)
Main professional activity, *n* (%)	55	
Crafts		49 (89.1)
Retired, *n* (%)	55	
Yes		33 (60)
Main source of income, *n* (%)	55	
Crafts		54 (98.2)
Pension / Retirement		32 (58.2)
Monthly income, based on the National Minimum Wage (NMW), *n* (%)	55	
Monthly income < NMW		35 (63.6)
Years of residence in the parish, M (SD)	55	56 (19.50)

Most participants were married (85.5%; *n* = 47), 9.1% (*n* = 5) were widowed, 3.6% (*n* = 2) divorced, and only 1.8% (*n* = 1) were single. The artisans’ mean years of education was 6.42 years (SD = 3.32), with a range of 0–19 years. The most expressive proportion of artisans had up to 4 years of education (43.6%, *n* = 24), while 18.2% (*n* = 10) had between 5 and 6 years, and 25.5% (*n* = 14) between 7 and 9 years of education. Almost a quarter of participants (23.6%, *n* = 13) reported having received professional training, with 11 different areas being identified, and one participant in each area (1.8%), except for the training-of-trainers area (5.5%, *n* = 3). Most participants (60%, *n* = 33) were retired, and for 89.1% (*n* = 49) the main professional activity was crafts and folk art. A minority (10.9%, *n* = 6) reported that craft activity was not their primary occupation. Almost all artisans (98.2%, *n* = 54) reported craft work as their main source of income, with 58.2% (*n* = 32) also referring to income from pension income, and 10.9% (*n* = 6) from other sources. The majority of artisans (63.6%, *n* = 35) placed their monthly income at or below the national minimum wage. Nearly all participants (96.4%; *n* = 53) lived in their own homes, and the mean length of residence in the parish was 56 years (SD = 19.50), with a range of 3–84 years.

### 4.2 Professional profile of older artisans

Most participants (52.7%, *n* = 29) work in the Imagery sector, while the remaining artisans are distributed among Pottery, Wood, Iron and Derivatives, Embroidery, Weaving, Basketry and Wicker and Contemporary Crafts. In the Embroidery sector, participants practiced the Crivo Embroidery of São Miguel da Carreira, characterized by delicate openwork on linen and recognized as intangible cultural heritage.

The age at which participants started at craft and folk arts ranged from 4 to 63 years, with a median of 12 years. Notably, 43.6% (*n* = 24) began practicing the activity before the age of 10, and 32.7% (*n* = 18) between 11 and 20 years old.

The majority of artisans (87.3%, *n* = 48) had their atelier located at their place of residence.

On average, artisans dedicated 51.24 h per week (SD = 17.05) to their craft activity. Weekly hours ranged from 18 to 96, with a median of 50 h. Most artisans (69.1%, *n* = 38) reported dedicating more than 40 h per week to craft activity. It was also observed that artisans spent an average of 10.05 h per week (SD = 9.33) on regularly performed activities outside their craftwork, with 15 different activities reported. The most prominent activities were household activities and subsistence farming (45.5%, *n* = 25), physical activity (36.4%, *n* = 20), and sociocultural activities (29.1%, *n* = 16). A total of 16.4% (*n* = 9) of participants reported not engaging in any regular activity outside their craftwork.

Regarding the days and times when craft activities are usually carried out, 92.7% (*n* = 51) reported working in the afternoon, 89.1% (*n* = 49) in the morning, from Monday to Friday, and 41.8% (*n* = 23) also reported working in the evening. The results further indicate that 65.5% (*n* = 36) work on Saturdays, and 30.9% (*n* = 17) occasionally on Sundays.

Different main raw materials used in the craft activity were reported (*n* = 12). Clay was by far the most highlighted material (65.5%; *n* = 36); iron, wood, and linen were mentioned by 9.1% (*n* = 5); wicker by 3.6% (*n* = 2), and the same percentage was observed for other raw materials: paper, aluminum, glazed material, and in this category, a minority mentioned pine cones, cotton, bamboo, stones, and other natural elements (1.8%; *n* = 1).

Participants reported the main thematic areas represented in the artistic activity, with 22 different areas being mentioned. Most artisans (78.2%, *n* = 43) mentioned the development of themes according to customer requests; 67.3% (*n* = 37) referred work on religion and festivals themes, and 52.7% (*n* = 29) mentioned the Barcelos Rooster (Galo de Barcelos). Also, 47.3% (*n* = 26) mentioned working on figures, miniatures; 27.3% (*n* = 15) on the animal world; 25.5% (*n* = 14) on scenes from everyday life; 16.4% (*n* = 9) on utilitarian pieces; 14.5% (*n* = 8) on devils and ambiguous figures; 7.3% (*n* = 4) on embroidery, 5.5% (*n* = 3) on Crivo Embroidery of São Miguel da Carreira and the same percentage on social criticism. A minority mentioned other areas, with 3.6% (*n* = 2) of participants indicating each of the following: flowers, personalities, pottery decoration, baskets, linen nativity scenes, and antiques restoration; while 1.8% (*n* = 1) indicated each of the following: artistic ceramics, caricatures, lamps, wooden toys, and flower painting. Table 2 describes the characterization of the artisans’ professional profile.

**Table 2:** Professional profile of the artisans.

Variables	*N*	Descriptive statistics
Craft sector, *n* (%)	55	
Imagery		29 (52.7)
Pottery		7 (12.7)
Wood		5 (9.1)
Iron and derivatives		4 (7.3)
Embroidery		3 (5.5)
Contemporary crafts		3 (5.5)
Weaving		2 (3.6)
Basketry and wicker		2 (3.6)
Age of entry into the craft sector, *n* (%)	55	
Up to 10 years		24 (43.6)
11–20 years		18 (32.7)
21–40 years		4 (7.3)
41–60 years		8 (14.6)
Over 60 years		1 (1.8)
Weekly hours dedicated to craft activity, M (SD)	55	51.24 (17.05)
Weekly hours dedicated to craft activity, *n* (%)	55	
Up to 20 h		3 (5.5)
21–40 h		14 (25.5)
41–60 h		27 (49.1)
61–80 h		8 (14.5)
Over 80 h		3 (5.5)
Weekly hours dedicated to non-professional activities, M (SD)	55	10.05 (9.33)
Days of craft activity, *n* (%)	55	
Monday to Friday		55 (100)
Saturday		36 (65.5)
Sunday (occasionally)		17 (30.9)
Atelier location, *n* (%)	55	
At home		48 (87.3)
Main raw materials used, *n* (%)	55	
Clay		36 (65.5)
Iron		5 (9.1)
Wood		5 (9.1)
Linen		5 (9.1)
Main themes represented, *n* (%)	55	
Theme as requested		43 (78.2)
Religion and festivity		37 (67.3)
Barcelos Rooster (Galo de Barcelos)		29 (52.7)

### 4.3 Transmission of craft practices and family work dynamics

Most artisans (63.6%; *n* = 35) stated that the legacy of craft and folk art was passed down by family members from previous generations.

The transmission was found to have occurred through various family members ascendants, collaterals, and relatives by affinity, with the highest frequency observed among ascendant relatives: grandmothers (36.4%; *n* = 20), grandfathers (34.5%; *n* = 19), fathers (32.7%; *n* = 18), and mothers (30.9%; *n* = 17). A minority (3.6%; *n* = 2) mentioned a collateral relative (uncle or aunt) and a relative by affinity (spouse). One participant (1.8%) referred to an earlier generational transmission from a great-grandmother. Group analysis highlighted certain artistic sectors as more prevalent in the transmission of the craft legacy, with the majority of cases concentrated in Pottery (100%, *n* = 7), Wood (80%, *n* = 4), Imagery (69%, *n* = 20), and the Embroidery (66.7%; *n* = 2). In contrast, no participants from the Iron and derivates or Basketry and wicker sectors reported having inherited the craft from family members of previous generations.

Regarding the period of initiation of craft activity within the family most artisans (54.5%; *n* = 30) reported that the activity began within their family in the 19th century. The second most frequently mentioned time period was the second half of the 20th century (20%; *n* = 11).

By craft sector, the majority of activities in the following sectors were identified as having begun within the family in the 19th century: Imagery (58.6%; *n* = 17), Pottery (85.7%; *n* = 6), Crivo Embroidery of São Miguel da Carreira (66.7%; *n* = 2), Wood (60%; *n* = 3), and Weaving (100%; *n* = 2).

The number of generations involved in the crafts and folk art sector ranged from 1 to 6. One generation was most frequently reported within the Contemporary Crafts sector (66.7%; *n* = 2), while six generations was mentioned by only one participant from the Crivo Embroidery of São Miguel da Carreira sector (33.3%; *n* = 1). The majority of artisans in the Pottery, Imagery, Crivo Embroidery of São Miguel da Carreira, Weaving, and Wood sectors reported that the craft has been practiced within the family for three or more generations.

A total of 78.2% (*n* = 43) of the artisans reported that they are transmitting their craft to younger generations and/or to the community. Among these, family members were most frequently mentioned, with a particular emphasis on children (52.7%; *n* = 29) and grandchildren (21.8%; *n* = 12). Transmission to community members was also reported by 21.8% of the artisans (*n* = 12). Most artisans (70.9%; *n* = 39) stated that they have family members working in the crafts sector. Among them, 61.5% (*n* = 24) reported having younger family members involved in the activity, 20.5% (*n* = 8) reported both older and younger family members, and 17.9% (*n* = 7) reported only older family members. Additionally, 29.1% (*n* = 16) of the participating artisans have no family members working in the crafts sector.

Among the participants who reported regularly working with other artisans, the same proportion was observed for collaboration with other generations and with the same generation (30.9%; *n* = 17). A total of 38.2% (*n* = 21) of participants reported working alone.

Of those who carried out the activity with the same or other generations (*n* = 34), collateral family members (siblings) and family members by affinity (husband, wife) were prevalent (58.8%, *n* = 20).

Regarding the number of family members involved in craft activities, a range from 1 to 8 individuals was observed, with a median of 2. Table 3 presents the data on family transmission and work dynamics in craft and folk art activity.

**Table 3:** Family transmission and work dynamics in craft and folk art activity.

Variables	*N*	Descriptive statistics
Transmitted by family members from previous generations, *n* (%)	55	35 (63.6)
Family members who transmitted the craft, *n* (%)	35	
Grandmother and grandfather		39 (70.9)
Father and mother		35 (63.6)
Other family members mentioned: uncle/aunt; spouse; great-grandmother		5 (9)
Reported period when the activity began in the family, *n* (%)	55	
19th century		30 (54.5)
Transmission to younger generations and/or the community, *n* (%)	55	43 (78.2)
Children		29 (52.7)
Grandchildren		12 (21.8)
Transmission to the community		12 (21.8)
Reported generations involved up to the present, *n* (%)	55	
3 or more generations		34 (61,9)
Reported family members involved in crafts, *n* (%)	55	39 (70.9)
Reported family members involved in crafts, by age group, *n* (%)	39	
Younger family members		24 (61.5)
Older family members		7 (17.9)
Both younger and older family members		8 (20.5)
Number of family members involved in crafts, Md	55	2
Regular engagement in craft work across generations, *n* (%)	55	
Yes, with other generations		17 (30.9)
Yes, with the same generation		17 (30.9)
Works alone		21 (38.2)
Regular engagement in craft work across generations, by family relationship, *n* (%)	34	
Collateral and affinal relatives (siblings, husband, wife)		20 (58.8)
Descendant relatives		13 (38.2)
Ascendant relatives		1 (2.9)

### 4.4 Associations between sociodemographic and professional characteristics of older artisans

Age was positively correlated with the number of years of residence in the parish (*n* = 55; r_s_ = 0.631; *p* < 0.001), and negatively correlated with years of education (*n* = 55; r_s_ = −0.573; *p* < 0.001), with the number of weekly hours dedicated to non-professional activities (*n* = 55; r_s_ = −0.462; *p* < 0.001), and with the age at which the activity in the crafts sector was initiated (*n* = 55; r_s_ = −0.290; *p* < 0.032).

A positive correlation was found between the years of education and the age at which the activity in the crafts sector was initiated (*n* = 55; r_s_ = 0.338; *p* = 0.012) and with the number of weekly hours dedicated to activities regularly performed outside of professional work (*n* = 55; r_s_ = 0.365; *p* < 0.006).

Women engaged in significantly more household activities during their non-professional time [χ^2^(1) = 28.171; *p* < 0.001], while in cultural activities they were less likely to report such involvement compared to men [χ^2^(1) = 4.490; *p* = 0.034].

A small difference was observed in the comparison of mean hours dedicated to craft activity between retired and non-retired individuals (M = 52.05, SD = 18.656; M = 50.70, SD = 16.172). Among the activities regularly performed outside of professional activity, a significant association was found between retirement status and caregiving activity (χ^2^ = 6.471, *p* = 0.021), indicating that non-retired participants are more likely to engage in this activity.

Although the results indicate that the mean age is slightly higher in the lower-income group (M = 68.23; SD = 7.535) compared to the higher-income group (M = 66.20; SD = 8.877), the tests performed showed no significant difference between the groups (*F* = 0.414; *p* = 0.523; t = 0.900; *p* = 0.186).

No statistically significant difference was also found for the number of weekly hours dedicated to the activity between the groups with different income levels. The results indicate that the mean number of hours dedicated is very similar (M = 51.66, SD = 19.73); (M = 50.50, SD = 11.32).

Regarding the activities performed outside of professional activity, the Chi-square test showed a marginally significant trend in caregiving (*p* = 0.049) and participation in household activities (*p* = 0.025), both more frequently reported in the Imagery sector.

Additionally, a significant negative correlation was observed between the number of family members involved in the activity and the age at which the individual began working in the crafts sector (r_s_ = −0.328; *p* < 0.05). Table 4 presents the associations between sociodemographic characteristics, variables related to professional activity, and variables related to family dynamics.

**Table 4:** Associations between sociodemographic characteristics and variables related to professional activity and family dynamics.

Variables		Statistical tests	p*
Age	Weekly hours dedicated to non-professional activities	*n* = 55; r_s_ = −0.462	<0.001
	Age of entry into the craft sector	*n* = 55; r_s_ = −0.290	<0.032
Gender: female	Non-professional activities: household activities	χ^2^ (1) = 28.171	<0.001
	Non-professional activities: cultural activities	χ^2^(1) = 4.490	0.034
Years of education	Weekly hours dedicated to non-professional activities	*n* = 55; r_s_ = 0.365	<0.006
	Age of entry into the craft sector	*n* = 55; r_s_ = 0.338	0.012
Retired	Non-professional activities: caregiving	χ^2^ = 6.471 (NR)	0.021
Number of family members involved in crafts	Age of entry into the craft sector	r_s_ = −0,328	0.05

*p < 0.05. *n*, Number of participants; rₛ, Spearman’s Rank Correlation Coefficient; χ^2^, Chi-square Test; p, p-value; NR, Not Retired.

## 5 Discussion

This cross-sectional study offers a characterization of the sociodemographic and professional profiles of older artisans performing their activity in a UNESCO Creative City of Crafts and Folk Art, contributing to emphasizing the sociocultural relevance of this practice. In addition, it explores the dynamics of family transmission of crafts and folk art within the specific context of Barcelos (Portugal), while highlighting the practice of craftsmanship as an example of active and productive aging. It stresses the importance of this activity not only as an occupation, but also as a form of cultural expression that promotes the transmission of knowledge both intergenerationally and within the community. These results may provide valuable insights for public policies, presenting empirical data that can contribute to evidence-based decision-making. They further demonstrate how craft and folk art activities can be framed as a strategy for promoting active and productive aging, representing one of the broader implications of this study, with potential impact on the enhancement of cultural heritage, the socioeconomic development of the community, and the formulation and transformation of policies related to culture, aging, and social inclusion (World Health Organization, 2020, 2021; United Nations, 2023; UNESCO, 2025). In this sense, the results intersect with current debates on cultural and social policies, potentially informing processes of development, evaluation, or transformation of public policies that foster the active participation of older adults in creative practices and the strengthening of cultural heritage. This study contributes to the literature that underscores not only the scarcity of research on spontaneous artistic activities carried out by older adults, but also the rarity of studies specifically focused on crafts and folk arts (Tzanidaki and Reynolds, 2011; Liddle et al., 2013; Fraser et al., 2015; Curtis et al., 2018; Pöllänen and Weissmann-Hanski, 2020; Chacur et al., 2022; Pesata et al., 2022). Additionally, it helps to address gaps in the research on family businesses (Porfírio et al., 2020; López-Chávez et al., 2021).

The studied sample has an average age of 67.49 years, above the standard retirement age in general and in Portugal, in particular (Lopes and Gonçalves, 2012; European Commission, 2025). However, the results indicate a strong dedication to crafts, with an average of over 50 weekly hours. This number contrasts with the average time spent on other activities regularly performed outside the craft context (M = 10.05).

The dedication to crafts is also reflected in the organization of working time, as in addition to weekly activities, most artisans continue to practice their craft on weekends. Thus, it is observed that, despite most participants being formally retired, many continue to work. These findings align with the literature on artisans in the region, which, in addition to highlighting the activities of older adults, also refers to the intense work and continuous involvement in this practice throughout life (Costa, 1991; Município de Barcelos, 2015; Costa, 2024).

Another relevant aspect is the early age of initiation into crafts. The majority began working in this field before the age of 20, and 43.6% (*n* = 24) started before the age of 10. In addition, this group shows, on average, fewer years of formal education and dedicates less time to non-craft activities, suggesting that, as in other professions, entry into crafts used to occur at an earlier age than it does today. On the other hand, younger participants tend to start their craft activity at a later age and have higher levels of formal education, which may be linked to the increase in mandatory schooling over recent decades. Furthermore, these artisans devote more weekly hours to non-professional activities, suggesting that higher educational attainment may be associated with a diversification of occupations and interests throughout life.

The analysis also reveals that nearly all participants live in their own homes within the territory, with the average number of years residing in the parish (M = 56) indicating long-term permanence in the same locality, suggesting a strong connection to place.

The study highlights a clear trend: older artisans began their craft at a younger age, have lived in the territory for longer periods, and tend to remain in the same locality as they age. These factors may be associated with residential stability, rootedness in the socio-cultural territory, and the strengthening of family and community ties.

The analysis reveals several significant correlations between sociodemographic and professional variables of the participants, reflecting associations between age, education, and engagement in craft activity.

The study did not reveal substantial differences in the number of weekly hours dedicated to craft activities among retired and non-retired artisans, with both demonstrating a high level of engagement in the activity. However, a statistically significant association was observed in caregiving roles, with non-retired participants being more likely to perform this role in activities outside the professional context.

Another relevant factor is the income generated from craft activity, which was identified by nearly all participants as their main source of livelihood, even though in most cases it falls below the national minimum wage. Another study, conducted with the same sample, examined the association between sociodemographic and professional characteristics and perceptions of health, quality of life, and happiness, and identified significant associations with these dimensions. In particular, higher income levels were associated with more positive perceptions of quality of life and facets of the environment domain (Igreja et al., 2025). These findings underscore the importance of income for quality of life among aging populations and highlight the need for public policies that improve the economic conditions of these professionals (Costa et al., 2018; Tavares, 2022). Increasing funding for creative activities has been identified as an effective strategy for promoting well-being on a large scale (Keyes et al., 2024).

Regarding non-professional activities regularly performed, women showed a significantly higher propensity to engage in domestic tasks and a lower level of involvement in cultural activities compared to men. Based on this data, differences in the time dedicated to craft work between men and women were expected but not confirmed. This outcome suggests the presence of different behavioral patterns, where the burden of domestic responsibilities may limit women’s participation in other occupations. These findings align with previous studies that examined gender issues within the Imagery sector in this region. Such studies indicate that clay-related activities have persisted largely due to family support, and that it was common for both men and women to work with clay. However, women often alternated between this activity and domestic responsibilities, including childcare (Cruz, 2004; Fernandes, 2024). The present results suggest that this dynamic may still be in place.

Most participants belong to the Imagery sector, followed by pottery, which is the second most represented in the sample. Although various raw materials such as clay, linen, and wood were reported, clay stood out as the most commonly used, reflecting the prominence of certain craft sectors within the region. Despite the existing artisanal diversity, Pottery and Imagery remain dominant (Correia, 1965; Município de Barcelos, 2015, 2023; Fernandes, 2024; UNESCO, 2024). Among the central themes in the artisans’ work are creations related to religion and festivals, as well as the Barcelos Rooster (Galo de Barcelos), a figure associated with justice and faith, linked to the legend of a pilgrim saved from death on the way to Santiago de Compostela. These elements align with the broader narrative about Barcelos, which, in addition to highlighting crafts and folk art as a distinguishing feature of local culture, points to products that have achieved wide recognition (Município de Barcelos, 2023; UNESCO, 2024). Additionally, the themes mentioned are present across various artisanal sectors represented in the sample, including Imagery, Pottery, Embroidery, Weaving, Iron and Derivatives, Wood, Basketry and Wicker, as well as Contemporary Crafts. The territorial context, characterized by a strong regional tradition in crafts and folk art, municipal support initiatives, and recognition by UNESCO as a Creative City of Crafts and Folk Art stands out (Município de Barcelos, 2023; UNESCO, 2024). These factors may contribute to a context conducive to active aging, supporting the continuation of creative craft practice. This study aligns with previous research demonstrating that the ability to be creative and productive is a central aspect of aging experiences (Gallistl, 2018).

Regarding the transmission of artisanal activity, most participants reported having inherited the legacy of crafts and folk art from older family members, particularly grandparents and parents. This finding suggests a connection with the early age at which they began practicing, reflecting early exposure to older relatives engaged in the craft.

The results also showed that most artisans report passing on their knowledge to younger generations, predominantly within the family but also in the community context. This highlights the importance of transmitting artisanal knowledge. This study aligns with previous research that has highlighted the role of family-based craft businesses passed down through generations, sustained by local culture and traditions, strengthening the way of life in rural areas and contributing to the local economy by promoting traditions (Deb et al., 2022).

These findings further underscore the role of older adults as custodians of knowledge and facilitators of continuity in craftsmanship, emphasizing their generative contribution to the perpetuation of this practice and their active role in preserving this sociocultural legacy. This study can add to previous research focused on creative practices among older adults (Gallistl, 2018) and broaden the scope of gerontological research by considering crafts and folk art as examples of active and productive aging.

The data indicate that most participants trace the origins of their craft activity within their families back to the 19th century, with particular emphasis on the sectors of Imagery, Pottery, Crivo Embroidery of São Miguel da Carreira, Weaving, and Wood. However, this temporal marker may reflect the limits of oral and family memory, being more closely related to available recollections than to the actual beginnings of these practices. It is plausible that many of these crafts have even earlier origins, preserved through informal knowledge transfer and direct observation, often transmitted in undocumented ways and passed down from generation to generation. This continuity highlights the importance of oral tradition and hands-on learning in the preservation and perpetuation of such crafts. These findings are consistent with the literature, which notes that by the 19th century, there were numerous references to pottery produced in the Barcelos region, whose ateliers became nationally recognized (Correia, 1965; Peixoto, 1990; Leal, 2002; Município de Barcelos, 2023; Leal, 2024).

Family transmission across three or more generations stands out in various sectors of crafts and folk art, highlighting the importance of craft family businesses in preserving local cultural heritage. The variation in the number of generations involved, ranging from one to six, reflects both the deep-rooted continuity in certain sectors, such as Crivo Embroidery and Pottery, and the relative novelty of others, such as Contemporary Crafts. These findings underscore the importance of cultural heritage and the role of families in the preservation and sustainability of these artisanal practices.

In addition to intergenerational transmission, most artisans report having family members working in the sector, with younger relatives standing out.

Work is regularly carried out both with members of the same generation and with those from different generations. It was also found that, in the regular performance of the work, collateral and affinity family members (siblings, husband, wife) are predominant, suggesting that their presence may be a facilitating factor in the continuity of craft practices by providing support and knowledge sharing, especially in a context where intergenerational transmission and early initiation into the craft have been widely observed.

Furthermore, the negative correlation observed between the number of family members involved in the craft and the age at which individuals began practicing suggests that greater family involvement provides more opportunities for early exposure to and familiarization with the craft. This finding highlights the role of families in the transmission of artisanal knowledge and in fostering intergenerational contact. These data reinforce the importance of family involvement in preserving cultural heritage and underscore the need for public policies that, on the one hand, promote education and training in these arts and, on the other hand, encourage family involvement and community support for the preservation and continuity of crafts and folk art. This family involvement also contributes to active and productive aging by strengthening support networks, participation, and a sense of purpose throughout the life course (Bárrios, 2015; Paúl and Lopes, 2016; Hijas-Gómez et al., 2020; World Health Organization, 2020, 2021; United Nations, 2023).

The promotion of the Sustainable Development Goals through family-run arts and crafts businesses is also essential, as these goals advocate, among other aspects, for the development of policies that support productive activities, creativity, and innovation (Nações Unidas, 2018). Such measures contribute to the economic viability and sustainability of these practices across generations. The support of local authorities can play a decisive role in this process, alongside efforts to promote this cultural tradition among tourists (Deb et al., 2022).

This study, composed of individuals whose daily lives are strongly shaped by craft activity, appears to align with findings from previous research showing that craft practice can foster a greater sense of purpose, belonging, and cultural and social awareness (Pöllänen, 2013; Pöllänen and Weissmann-Hanski, 2020; Keyes et al., 2024). Furthermore, by highlighting the engagement of older individuals in meaningful cultural and economic activities within their local territory, the data reinforce crafts as an example of active aging. In the previous study with the same sample, the majority of artisans perceived their overall quality of life as “good,” reported feeling “very happy,” and stated they were satisfied or very satisfied with their health (Igreja et al., 2025). Considering a life course perspective, most artisans began their craft at an early age, keep being productive during midlife and it is interesting to note that 60% continue their practice after retirement age, highlighting a life journey dedicated to crafts and folk art. The integration of the personal and interpersonal dimensions, as a fundamental process of human development, enables the attainment of wisdom through integrity, understood as the most evolved quality of the self (Novo, 2000). Old age, understood as the crowning of the entire sequence of experiences from earlier stages, brings with the existential integrity, built stage by stage and essential for continuing onward (Paúl, 1991).

The results of this study suggest that participants may be reaching this state of wisdom. Beyond the individual dimension, the data also suggest manifestations of family integrity, which can be experienced as a deep and enduring sense of peace and/or satisfaction with one’s multigenerational family relationships—past, present, and future (King and Wynne, 2004). Additionally, these results seem to align with studies on active aging, which highlighted, among other factors, motivations for involvement related to local traditions (Wongsala et al., 2021). Craftwork emerges as a relevant example of active and productive aging, where its continued practice within the family context highlights its potential contribution to well-being, social participation, and the preservation of cultural heritage. By analyzing this evidence in light of the UNESCO recommendations for Creative Cities (UNESCO, 2025) and the international strategies for active and healthy aging (United Nations, 2020; World Health Organization, 2020, 2021; United Nations, 2023), this study positions itself within ongoing academic and practical discourses, reinforcing the need for interdisciplinary approaches that unite culture, active and healthy aging, and community development. For example, the finding that more than 60% of artisans continue their activity after retirement highlights the potential of craftwork as a model of active and healthy aging. This aligns with the emphasis of the World Health Organization (2020, 2021) and the United Nations (2020, 2023) on social participation and well-being throughout the life course, reinforcing the importance of creating conditions that enable older people to continue contributing to their families, communities, and economies. Likewise, the strong presence of multigenerational family transmission, in several sectors involving three or more generations, underscores the central role of family-based craft businesses in preserving local cultural heritage and strengthening the economy. This is in line with UNESCO’s recommendation (UNESCO, 2025) to promote creativity and cultural industries, valuing culture as a driver of sustainable community development. These findings are also in line with national priorities outlined in the strategic pillars of the Active and Healthy Aging Action Plan, which aims to maximize quality longevity, ensure the maintenance of autonomy, enhance quality of life, and promote the creation of economic and social opportunities in an evolving society (Presidência do Conselho de Ministros, 2024).

This study may contribute to the development of public policies that are applicable across different sectors, benefiting all artisans. Such measures may include the economic valorization of handcrafted pieces, ensuring a fair price that reflects the time, dedication, and uniqueness of each artisan’s work; structured support for the commercialization of products; the provision of specialized training tailored to the needs of artisans; business management education; and the promotion of intergenerational initiatives that encourage the engagement of both older and younger artisans, thereby fostering the sustainability of these practices within the territory. These actions may support active and productive aging, aligning with studies that highlight the importance of considering factors such as community culture when designing programs aimed at promoting active aging (Shahla et al., 2023). Grounded in traditional crafts and folk art, practices intrinsically linked to the sociocultural identity of the participants, these findings provide insights that may inform the development of initiatives and public policies in this field. Moreover, this evidence gains even greater importance in light of demographic trends, which pose urgent challenges for the definition of effective policies. This is particularly relevant given the ongoing demographic shift in Portugal, as evidenced by indicators such as the aging index, the rejuvenation index of the active population, and the potential sustainability index. According to the latest Census, the percentage of older people (65 and over) stood at 23.4%, while that of young people (aged 0–14) was only 12.9% (Instituto Nacional de Estatística, 2022). In this way, the study offers contributions to and incentivizes international academic and policy debates on active aging and cultural policies, aligning with global guidelines and strategies (UNESCO, 2025; United Nations, 2020; World Health Organization, 2020, 2021; United Nations, 2023).

## 6 Conclusion

These findings are relevant in the context of active aging and the perspective of aging as a life course process. In this particular case active aging appears in the continuity of earlier phases of development, showing people maintaining their participation in professional activities that are valued by the community and that seems to contribute to self-esteem and quality of life. The municipality of Barcelos gives emphasis to these art and crafts, valuing the artisans and stimulating the preservation and transmission of their unique knowledge as a legacy for future generations.

This study, in addition to characterizing the sociodemographic and professional profiles of participants, explores the family transmission dynamics of older artisans practicing in Barcelos, a UNESCO Creative City in the field of Crafts and Folk Art. It highlights craftsmanship as a representative practice of active and productive aging. The research contributes to the existing literature on these art forms by focusing on activities within the crafts and folk art sector carried out spontaneously by older individuals, and by presenting findings on family transmission processes.

From a broader perspective we learned that the activity began within the family mostly in the 19th century. The practice was passed down by relatives from previous generations, particularly parents and grandparents, with three or more generations involved up to the present day. However, this temporal milestone may reflect the limits of oral and family memory, not excluding the possibility of older practices. On the other hand, the transmission of knowledge to younger generations or to the community was mentioned by a large number of artisans. Furthermore, regular involvement in the craft predominantly occurs among collateral relatives (siblings) and affinal relatives (husband or wife). These findings emphasize the importance of family transmission and involvement in the preservation of craftsmanship, highlighting the relevance of public policies that support and promote the recognition of older artisans, encourage the intergenerational transmission of craftsmanship, and the long-term sustainability of family businesses, reinforcing the preservation of cultural heritage.

Although this study provides comprehensive data on the sociodemographic and professional profiles of older artisans in Barcelos, as well as on the family transmission dynamics of craftsmanship, highlighting it as an example of active and productive aging, it is important to acknowledge and discuss its limitations. The sample size is one such limitation. Future research could benefit from a larger sample and the inclusion of younger professional artisans from different sectors, which would allow for a broader understanding of sociodemographic profiles, professional trajectories, and family dynamics.

The absence of a control group composed of older individuals from the same region with different or no occupations limited the ability to compare aspects of the aging process. On the other hand, the results highlight the relevance of further investigating factors such as the transgenerational legacy of craftsmanship and the generativity associated with artistic practice. Although the results cannot be generalized, it is important to note that the sample represents nearly 30% of the artisans in the region, providing a valuable insight into this population. Despite these limitations, the data obtained in this study provide important starting points for future empirical research. Further research integrating both qualitative and quantitative approaches could explore contextual and psychosocial aspects, as well as other factors perceived by men and women, including family functioning dimensions. Such studies may provide more robust data regarding craft and folk art activities and their family dynamics, particularly in relation to active aging.

The study aligns with the global agenda for promoting a more equitable, inclusive, and sustainable society, emphasizing the need to improve the lives of older individuals, their families, and the communities in which they live. This commitment aims to ensure that they can realize their potential with dignity and equality in a healthy environment. Furthermore, the study also connects with national and international strategies for active and healthy aging, contributing relevant information to combat ageism (Nações Unidas, 2018; United Nations, 2020; World Health Organization, 2020, 2021; United Nations, 2023; Presidência do Conselho de Ministros, 2024). It emphasizes positive concepts associated with aging, contributing to the recognition of older individuals by highlighting their active participation in creative activities, not only as passive participants but also as promoters of activity, underscoring their central role in the economic and sociocultural dynamics of the family and community. The study may provide a scientific contribution to active and healthy aging programs (Nações Unidas, 2018; World Health Organization, 2020, 2021). Thus, by describing the profiles of artisans and the dynamics of family transmission, the results provide evidence that can support the development, evaluation, or transformation of public policies, strengthening strategies that integrate culture, active aging, and community development.

## Data Availability

The raw data supporting the conclusions of this article will be made available by the authors, without undue reservation.
