# Supplementary material for: Crafts as an example of active and productive aging: profiles of artisans and family transmission in a UNESCO Creative City of Crafts and Folk Art
Source: Front Psychol. 2025 Aug 29;16:1651086. doi: 10.3389/fpsyg.2025.1651086 (PMC12426242; doi:10.3389/fpsyg.2025.1651086)
Supplement: Supplementary file 1 [file Data_Sheet_1.pdf]

STROBE Statement—Checklist of items that should be included in reports of ***cross-sectional studies***

	Item No	Recommendation	Section heading
**Title and abstract**	1	(*a*) Indicate the study's design with a commonly used term in the title or the abstract	Abstract
		(*b*) Provide in the abstract an informative and balanced summary of what was done and what was found	Abstract
**Introduction**			
Background/rationale	2	Explain the scientific background and rationale for the investigation being reported	1. Introduction; 2. Crafts and folk art: the legacy of Barcelos, Portugal
Objectives	3	State specific objectives, including any prespecified hypotheses	1. Introduction
**Methods**			
Study design	4	Present key elements of study design early in the paper	3.1 Study design
Setting	5	Describe the setting, locations, and relevant dates, including periods of recruitment, exposure, follow-up, and data collection	3.2 Participants and recruitment
Participants	6	(*a*) Give the eligibility criteria, and the sources and methods of selection of participants	3.2 Participants and recruitment
Variables	7	Clearly define all outcomes, exposures, predictors, potential confounders, and effect modifiers. Give diagnostic criteria, if applicable	3.3 Instruments
Data sources/ measurement	8*	For each variable of interest, give sources of data and details of methods of assessment (measurement). Describe comparability of assessment methods if there is more than one group	3.3 Instruments

Bias	9	Describe any efforts to address potential sources of bias	3. Materials and methods
Study size	10	Explain how the study size was arrived at	3.2 Participants and recruitment
Quantitative variables	11	Explain how quantitative variables were handled in the analyses. If applicable, describe which groupings were chosen and why	3.4 Data analysis, 3.4.1 Descriptive statistical analysis
Statistical methods	12	(*a*) Describe all statistical methods, including those used to control for confounding	3.4 Data analysis, 3.4.1 Descriptive statistical analysis
		(*b*) Describe any methods used to examine subgroups and interactions	N/A
		(*c*) Explain how missing data were addressed	N/A (no missing data)
		(*d*) If applicable, describe analytical methods taking account of sampling strategy	N/A
		(*e*) Describe any sensitivity analyses	N/A
**Results**			
Participants	13*	(a) Report numbers of individuals at each stage of study—eg numbers potentially eligible, examined for eligibility, confirmed eligible, included in the study, completing follow-up, and analysed	4. Results
		(b) Give reasons for non-participation at each stage	N/A
		(c) Consider use of a flow diagram	N/A
Descriptive data	14*	(a) Give characteristics of study participants (eg demographic, clinical, social) and information on exposures and potential confounders	4.1 Sociodemographic profile of older artisans; 4.2 Professional profile of older artisans
		(b) Indicate number of participants with missing data for each variable of interest	N/A (no missing data)
Outcome data	15*	Report numbers of outcome events or summary measures	4.2 Professional profile of older artisans; 4.3 Transmission of craft

			practices and family work dynamics
Main results	16	(*a*) Give unadjusted estimates and, if applicable, confounder-adjusted estimates and their precision (eg, 95% confidence interval). Make clear which confounders were adjusted for and why they were included	4.4 Associations between sociodemographic and professional characteristics of older artisans
		(*b*) Report category boundaries when continuous variables were categorized	4.4 Associations between sociodemographic and professional characteristics of older artisans
		(*c*) If relevant, consider translating estimates of relative risk into absolute risk for a meaningful time period	N/A
Other analyses	17	Report other analyses done—eg analyses of subgroups and interactions, and sensitivity analyses	N/A
**Discussion**			
Key results	18	Summarise key results with reference to study objectives	5. Discussion
Limitations	19	Discuss limitations of the study, taking into account sources of potential bias or imprecision. Discuss both direction and magnitude of any potential bias	6. Conclusion
Interpretation	20	Give a cautious overall interpretation of results considering objectives, limitations, multiplicity of analyses, results from similar studies, and other relevant evidence	5. Discussion; 6. Conclusion
Generalisability	21	Discuss the generalisability (external validity) of the study results	6. Conclusion
**Other information**			
Funding	22	Give the source of funding and the role of the funders for the present study and, if applicable, for the original	This study was self-funded by the first author as part of her Ph.D. studies. The publication fees for this article were supported by the

	study on which the present article is based	School of Medicine and Biomedical Sciences, University of Porto (ICBAS-UP), through the Doctoral Programme in Gerontology and Geriatrics.

*Give information separately for exposed and unexposed groups.

Note: An Explanation and Elaboration article discusses each checklist item and gives methodological background and published examples of transparent reporting. The STROBE checklist is best used in conjunction with this article (freely available on the Web sites of PLoS Medicine at http://www.plosmedicine.org/, Annals of Internal Medicine at http://www.annals.org/, and Epidemiology at http://www.epidem.com/). Information on the STROBE Initiative is available at www.strobe-statement.org.
